# A study on the clinical efficacy and safety of repetitive transcranial magnetic stimulation combined with transcranial direct current stimulation in adolescent depression

**DOI:** 10.3389/fpsyt.2025.1616641

**Published:** 2025-10-20

**Authors:** Qingyu Zhang, Lu Yang, Zhenzhen Zhu, Guanjun Wang, Jieqiong Hu, Maoqing Tong, Zhongze Lou, Ying Chai, Yun Ye, Yan Chen, Lanlan Wang, Kuilai Wang, Shuo Zhao, Ying Wu, Yanjie Li, Ke Yuan, Ziyi He, Yanbin Hou, Liemin Ruan, Yunxin Ji

**Affiliations:** ^1^ Department of Psychiatry and Behavioral Medicine, First Hospital Affiliated to Ningbo University, Ningbo, Zhejiang, China; ^2^ Department of Psychiatry, Kangning Hospital Affiliated to Ningbo University, Ningbo, Zhejiang, China; ^3^ Department of Psychiatry, Ningbo Kangning Hospital, Ningbo, Zhejiang, China; ^4^ School of Medicine, Ningbo University, Ningbo, Zhejiang, China

**Keywords:** adolescent depression, repetitive transcranial magnetic stimulation, transcranial direct current stimulation, combined treatment, efficacy

## Abstract

**Background:**

The prevalence of adolescent depression has been steadily rising, while the effectiveness of existing treatments remains limited, highlighting the urgent need for novel therapeutic strategies.

**Objective:**

To evaluate the efficacy and safety of combining repetitive transcranial magnetic stimulation (rTMS) with transcranial direct current stimulation (tDCS) in treating adolescent depression.

**Methods:**

This was a prospective, randomized, parallel-group controlled trial. A total of 260 hospitalized adolescents diagnosed with major depressive disorder (MDD) were enrolled and randomly assigned to one of four groups: G1 (medication only), G2 (medication + tDCS), G3 (medication + rTMS), and G4 (medication + combined tDCS and rTMS). Clinical assessments were conducted at baseline and after 4 weeks by trained evaluators blinded to group allocation. The primary efficacy outcome was the reduction rate in HAMD-17 scores. Secondary outcomes included changes in HAMA and PSQI scores.

**Results:**

Both the G3 and G4 groups showed significant improvements in depressive symptoms compared to G1 (*p* < 0.05 and *p* < 0.01, respectively), with anxiety symptoms also showing significant improvement (*p* < 0.05). However, no significant differences were observed for sleep quality improvement (*p* > 0.05). Regression analysis indicated that baseline depression severity and illness duration were key predictors of treatment response (*p* < 0.001). All interventions were well tolerated, and no serious adverse events were reported.

**Conclusion:**

The combination of rTMS and tDCS demonstrates superior efficacy over pharmacotherapy alone in reducing depressive symptoms in adolescents, with a favorable safety profile.

## 1 Introduction

Depression in adolescents is highly prevalent and has severe implications for health, making it a major global public health concern. Epidemiological studies indicate that the lifetime prevalence of Major Depressive Disorder (MDD) among adolescents ranges from 14% to 20% (1). Depression leads to substantial functional impairments in adolescents, impacting their academic performance, social relationships, and mental well-being, while also increasing the risks of substance abuse and suicide (2). For moderate to severe cases of adolescent depression, standard treatment typically includes a combination of antidepressant medications (e.g., SSRIs) and psychotherapy (3). However, many patients show inadequate responses to current treatments and continue to experience significant residual symptoms, underscoring the need for novel therapeutic strategies. Additionally, the efficacy and safety of antidepressants in adolescents remain subjects of ongoing debate (4). Hence, there is a pressing need for novel treatment strategies to enhance therapeutic efficacy. Rapid-acting biological interventions, such as ketamine infusion and electroconvulsive therapy (ECT), have shown promise, yet their invasiveness, resource requirements, and side-effect burden limit routine use in adolescents (5).

In recent years, non-invasive brain stimulation techniques have garnered increasing attention as potential treatments for depression. Repetitive transcranial magnetic stimulation (rTMS) and transcranial direct current stimulation (tDCS) are currently two of the widely recognized techniques. rTMS has been shown to be effective in treating adult depression and has received clinical approval for use (6). While rTMS is widely used in adults, research on its application in adolescents remains limited, although preliminary evidence suggests it is both effective and safe for this population (1). In recent years, tDCS has also been gradually incorporated into depression intervention studies. Although some studies support its antidepressant effects, its overall efficacy is considered more modest compared to rTMS, and in some large studies, no significant difference was found between tDCS and placebo stimulation (7). Currently, tDCS is more commonly regarded as an adjunctive treatment, particularly suitable for mild to moderate depression or as part of combined therapy (8). Recent optimization strategies for rTMS—including theta-burst stimulation, accelerated multi-session schedules, and deep-coil designs—aim to shorten session length while retaining or enhancing efficacy (9). Nevertheless, accelerated TMS schedules also have limitations: protocols remain heterogeneous and not yet standardized; the durability of benefit is still uncertain; safety profiles under higher cumulative dosing require further confirmation; and logistical demands (e.g., staffing and device time) may be substantial. Recent expert reviews emphasize that larger, rigorously controlled trials are needed before firm clinical practice recommendations can be made (10, 11). In addition, MRI-guided individualized targeting is being explored to tailor stimulation sites to each patient’s functional connectivity pattern, which may further improve response rates (12). However, randomized trials have yielded mixed results: for example, an RCT in recurrent depression found that MRI-guided coil positioning did not outperform the standard 5-cm method (13), and a recent multicenter, double-blind RCT similarly showed no superiority of MRI-based neuronavigation over the standard technique (14).

The distinct mechanisms of rTMS and tDCS suggest a synergistic effect when combined. rTMS activates neurons by using magnetic pulses, while tDCS modulates the membrane potential of neurons through electrical currents, leading to complementary effects that enhance therapeutic efficacy. Existing studies in adults support this hypothesis, demonstrating that the combination of rTMS and tDCS is more effective than either treatment alone (15), with comparable safety profiles for both methods.

However, research on combined brain stimulation treatments for adolescent depression is still limited. Given the unique characteristics of adolescent brain development, adolescents’ responses to rTMS and tDCS may differ from those observed in adults. As such, studying the efficacy and safety of adding rTMS and/or tDCS to pharmacological treatments for adolescent depression is of vital importance for expanding therapeutic strategies for this population. We hypothesized that the combination of pharmacological treatment with both rTMS and tDCS would result in greater symptom improvement than pharmacological treatment alone or in combination with either neuromodulation technique alone.

## 2 Materials and methods

### 2.1 Participants

This prospective randomized controlled trial enrolled 260 hospitalized adolescents diagnosed with major depressive disorder (MDD) between October 2021 and March 2023. All participants were recruited from the psychiatric inpatient services of the First Hospital Affiliated to Ningbo University.

**Inclusion criteria were as follows:**

(1) Age between 13 and 18 years;
(2) Meeting the diagnostic criteria for MDD according to the Diagnostic and Statistical Manual of Mental Disorders, Fifth Edition (DSM-5), confirmed by two board-certified psychiatrists;
(3) Receiving antidepressant treatment during hospitalization;
(4) Provision of written informed consent by both the patient and their legal guardian.

**Exclusion criteria included:**

(1) Diagnosis of bipolar disorder, schizophrenia, or other psychotic disorders;
(2) History of neurological disorders such as epilepsy, brain tumors, or traumatic brain injury;
(3) Substance or alcohol abuse within the past 6 months;
(4) Contraindications to rTMS or tDCS, such as implanted metallic devices;
(5) Acute suicidal risk requiring intensive intervention;
(6) Previous exposure to rTMS or tDCS within the past 6 months;
(7) Any changes to antidepressant regimen within 2 weeks prior to enrollment.

**Figure 1:**
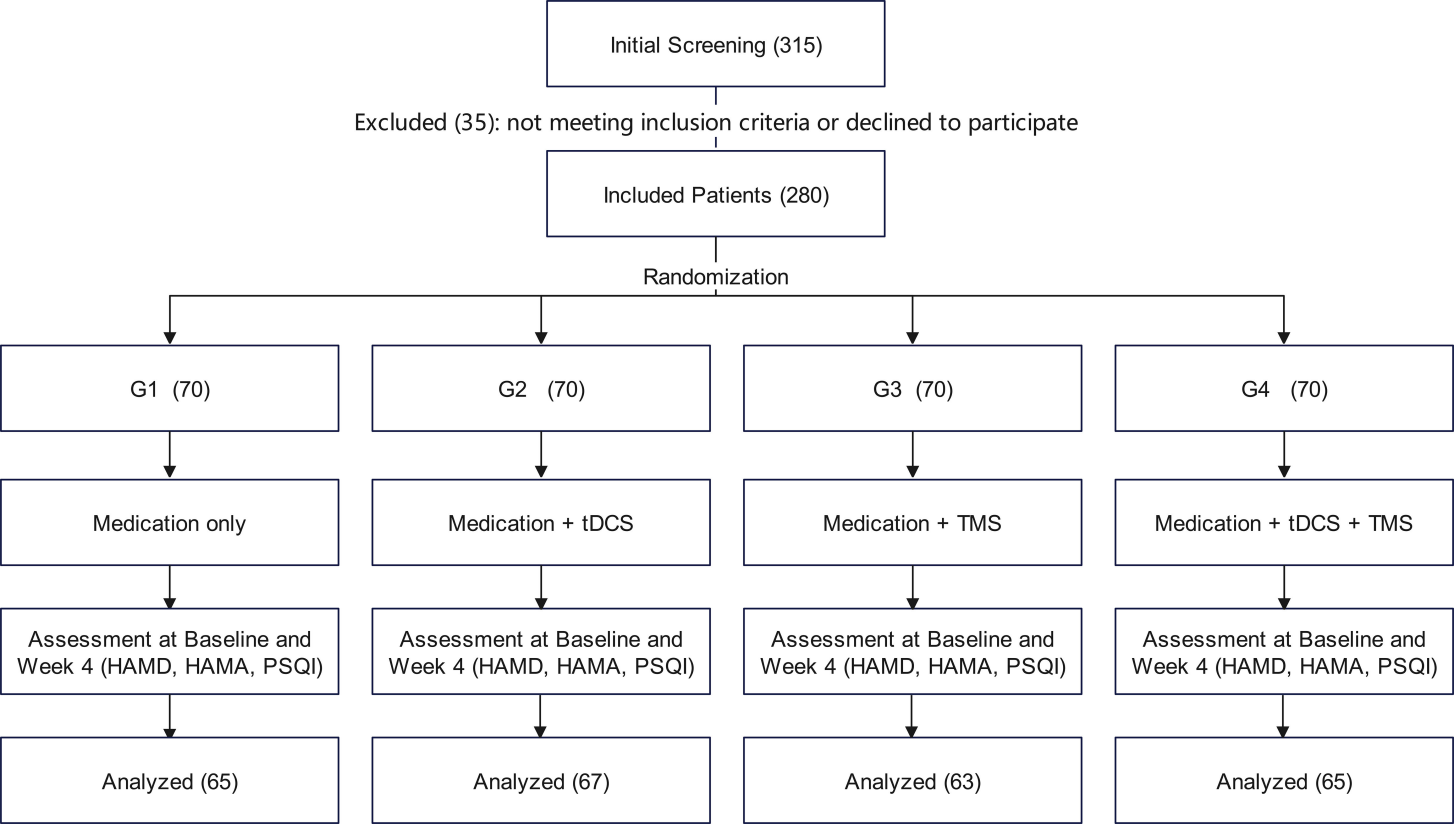
CONSORT flow diagram. HAMD, Hamilton Depression Rating Scale; HAMA, Hamilton Anxiety Rating Scale; PSQI, Pittsburgh Sleep Quality Index.

A total of 315 patients were initially screened. After excluding 35 patients who did not meet inclusion criteria or declined to participate, 280 were enrolled. Of these, 260 patients completed the full intervention protocol and were included in the final analysis (**Figure 1**).

### 2.2 Study design and randomization

This study was a prospective, randomized, parallel-group controlled trial designed to minimize bias and facilitate robust comparisons across treatment groups. Participants were randomly assigned to one of four treatment groups in a 1:1:1:1 ratio.

Randomization was conducted using a computer-generated random number table, prepared in advance by an independent statistician not involved in recruitment or assessment. Group assignments were sealed in opaque envelopes and opened sequentially after patient enrollment.

Due to the inherent differences in intervention methods, particularly the particularly the perceptible sensations associated with rTMS, complete blinding of both participants and operators was not feasible. However, outcome assessments were performed by trained evaluators who were blinded to treatment allocation, in order to reduce detection bias.

For ethical reasons, all participants received antidepressant medication during the study, and thus, a pure placebo control group was not included.

### 2.3 Intervention

All participants received standard antidepressant treatment. The prescribed medication was sertraline, a selective serotonin reuptake inhibitor (SSRI), administered at doses up to 200 mg/day. The exact dosage was determined by the study psychiatrist according to each participant’s clinical condition. Unless clinically indicated, the dosage remained stable throughout the 4-week treatment period.

Based on this foundation, the four participant groups received the following distinct interventions:

G1 (Medication-only): This group received only antidepressant medication alone, without any brain stimulation interventions.

G2 (Medication + tDCS): In addition to medication, patients in this group received tDCS during the first two weeks of treatment, five times per week for a total of 10 sessions. Stimulation was delivered to 2 mA, applied for 20 minutes, with the anode positioned on the left dorsolateral prefrontal cortex (DLPFC) and the cathode on the right DLPFC. Electrodes were positioned according to the international 10–20 EEG system at the F3 and F4 locations.

G3 (Medication + rTMS): Participants in this group received rTMS alongside medication treatment during the first two weeks, five times per week for a total of 10 sessions. rTMS was administered using a figure-eight coil over the left DLPFC, with a stimulation frequency of 10 Hz and an intensity of 100%-120% of the resting motor threshold, delivering 1600–2000 pulses per session.

G4 (Medication + rTMS + tDCS): In this group, both tDCS and rTMS interventions were applied sequentially during each treatment session. Stimulation parameters were identical to those in G2 and G3, with tDCS administered first, followed by prompt electrode removal and subsequent rTMS.

All interventions were conducted by professionally trained medical personnel under strict safety protocols. Throughout the treatment, researchers continuously monitored patient adherence and adverse events. If any patient experienced significant discomfort, the intervention could be paused or terminated at any point, as required.

### 2.4 Assessment indicators

Symptom assessment focused on three domains: depressive symptom severity was measured using the 17-item Hamilton Depression Rating Scale (HAMD-17), anxiety symptoms were assessed using the Hamilton Anxiety Rating Scale (HAMA), and sleep quality was self-reported by participants via the Pittsburgh Sleep Quality Index (PSQI). All scales were administered at both baseline (pre-treatment) and at the end of week 4. All evaluations were conducted by trained assessors who remained blinded to group allocation throughout the process to minimize observational bias.

In addition, demographic data (including age, sex, height, weight, and only-child status) and clinical characteristics (e.g., duration of illness and baseline scale scores) were collected for subsequent analysis.

Primary Efficacy Endpoint: The primary endpoint was the percentage reduction in HAMD-17 scores at week 4 calculated as: (baseline score − week 4 score)/baseline score × 100%. This measure reflects the relative improvement in depressive symptoms and helps control for baseline variability across patients.

Secondary Efficacy Endpoints: These included the percentage reduction in HAMA and PSQI scores, which assessed treatment-related improvements in anxiety symptoms and sleep quality, respectively, following treatment, respectively.

Analysis of Factors Influencing Efficacy: To assess the impact of baseline characteristics on treatment efficacy, a logistic regression model was used. A ≥25% reduction in HAMD scores was defined as “effective improvement”, which served as the dependent variable. Independent variables included treatment group (G2, G3, G4; G1 as the reference) and baseline characteristics such as gender, age, disease duration, BMI, and baseline HAMD score.

Adverse Event Monitoring: All adverse events (AEs) were documented following each treatment session or follow-up visit throughout the intervention period. Particular attention was paid to the common side effects of rTMS and tDCS, including headache, scalp tingling, dizziness, nausea, tremors, fatigue, drowsiness, and dry mouth. In the case of a serious adverse event (such as a seizure or suicidal behavior), the intervention would be immediately discontinued, and appropriate safety measures would be initiated.

### 2.5 Statistical analysis

All statistical analyses were performed using SPSS 25.0. Baseline characteristics between groups were compared using one-way analysis of variance (ANOVA) for continuous variables and chi-square tests (χ² tests) for categorical variables, to assess the comparability of the groups at baseline following randomization. For efficacy analysis, continuous outcome variables, such as the reduction rates in HAMD, HAMA, and PSQI scores, were compared using one-way ANOVA. If the overall differences were significant, pairwise comparisons between groups were conducted using the Bonferroni or Tukey HSD method. To explore potential factors affecting treatment response, a logistic regression model was constructed with “effective improvement” (defined as a ≥25% reduction in HAMD scores) as the dependent variable, and treatment group (dummy-coded) and baseline characteristics (gender, age, disease duration, BMI, and baseline HAMD score) as independent variables. Odds ratios (ORs) and 95% confidence intervals (CIs) were reported. All statistical tests were two-tailed, with a significance level set at *p* < 0.05. For analyses involving multiple comparisons, the Bonferroni method was used to adjust the significance level when necessary.

### 2.6 Ethics

The study protocol was approved by the Ethics Committee of the First Hospital Affiliated to Ningbo University (Approval number 2022-047A). The protocol was also registered with the National Medical Research Registry of China (Registration number MR-33-22-023081) and was publicly accessible via the Chinese Clinical Trial Registry at the time of registration. All participants and their legal guardians provided written, informed consent prior to enrollment, confirming their voluntary participation and full understanding of the study’s objectives, procedures, potential risks, and benefits. All research data were de-identified to ensure confidentiality and protection of patient privacy. The study was conducted in accordance with the principles of the Declaration of Helsinki and all relevant national and institutional ethical standards.

## 3 Results

### 3.1 Baseline characteristics

The sample sizes for each group were as follows: G1 (n=65), G2 (n=67), G3 (n=63), and G4 (n=65). **Table 1** presents the demographic and clinical baseline characteristics of the four groups. The average age of the patients was 14.1 ± 1.4 years, with females comprising 76.2%. The proportion of only children was 62.7%, and the mean disease duration was 11.7 ± 8.2 months. At baseline, the mean HAMD-17 score was 21.05 ± 5.92, with 56.9% of patients classified as having severe depression (HAMD ≥24), 37.3% with moderate depression, and 5.8% with mild depression. The baseline HAMA and PSQI scores were 22.86 ± 7.27 and 9.90 ± 3.93, respectively. There were no statistically significant differences between the groups in terms of age, gender, height, weight, BMI, family structure, disease duration, depression severity, or the baseline scores on the three main assessment scales (HAMD, HAMA, PSQI) (*p* > 0.05), indicating that the baseline characteristics of the groups were well balanced.

**Table 1:** Baseline demographic and clinical characteristics (Mean ± SD or n(%)).

Characteristic	Overall (n=260)	G1 (n=65)	G2 (n=67)	G3 (n=63)	G4 (n=65)	*p*
Age (years)	14.1 ± 1.4	14.1 ± 1.5	14.0 ± 1.3	14.2 ± 1.5	14.0 ± 1.4	0.93
Height (cm)	163.56 ± 7.44	163.2 ± 7.6	163.5 ± 7.2	164.1 ± 7.0	163.5 ± 7.9	0.88
Weight (kg)	56.11 ± 14.17	55.0 ± 12.8	56.6 ± 15.3	56.1 ± 13.9	56.7 ± 14.8	0.93
BMI (kg/m²)	20.87 ± 4.46	20.5 ± 4.2	21.1 ± 4.8	20.8 ± 4.5	21.0 ± 4.4	0.88
Gender [n(%)]						0.89†
Female	198 (76.2%)	51 (79.7%)	52 (77.6%)	46 (73.0%)	49 (74.2%)	
Male	62 (23.8%)	14 (21.5%)	15 (23.1%)	17 (26.2%)	16 (24.6%)	
Family Structure [n(%)]						0.37†
Only child	163 (62.7%)	38 (59.4%)	45 (67.2%)	35 (55.6%)	45 (68.2%)	
One sibling	83 (31.9%)	23 (35.4%)	17 (26.2%)	24 (36.9%)	19 (29.2%)	
Two siblings	14 (5.4%)	5 (7.7%)	5 (7.7%)	3 (4.6%)	1 (1.5%)	
Disease Duration (months)	11.74 ± 8.18	11.3 ± 8.5	11.9 ± 8.1	12.3 ± 8.4	11.6 ± 7.9	0.80
Depression Severity [n(%)]						0.86†
Severe	148 (56.9%)	34 (53.1%)	37 (55.2%)	36 (57.1%)	41 (62.1%)	
Moderate	97 (37.3%)	23 (35.4%)	26 (40.0%)	27 (41.5%)	21 (32.3%)	
Mild	15 (5.8%)	5 (7.7%)	3 (4.6%)	4 (6.2%)	3 (4.6%)	
HAMD Baseline	21.05 ± 5.92	21.3 ± 6.2	20.8 ± 5.4	20.4 ± 5.7	21.7 ± 6.3	0.62
HAMA Baseline	22.86 ± 7.27	23.2 ± 7.2	22.9 ± 7.7	21.8 ± 6.2	23.5 ± 7.4	0.60
PSQI Baseline	9.90 ± 3.93	9.52 ± 3.86	9.37 ± 3.99	9.87 ± 3.83	10.83 ± 3.83	0.14

*p*-values were determined using one-way ANOVA or Kruskal-Wallis tests for group comparisons; † denotes chi-square test. G1: Medication; G2: Medication + tDCS; G3: Medication + rTMS; G4: Medication + tDCS + rTMS.

### 3.2 Primary endpoint

**Figure 2:**
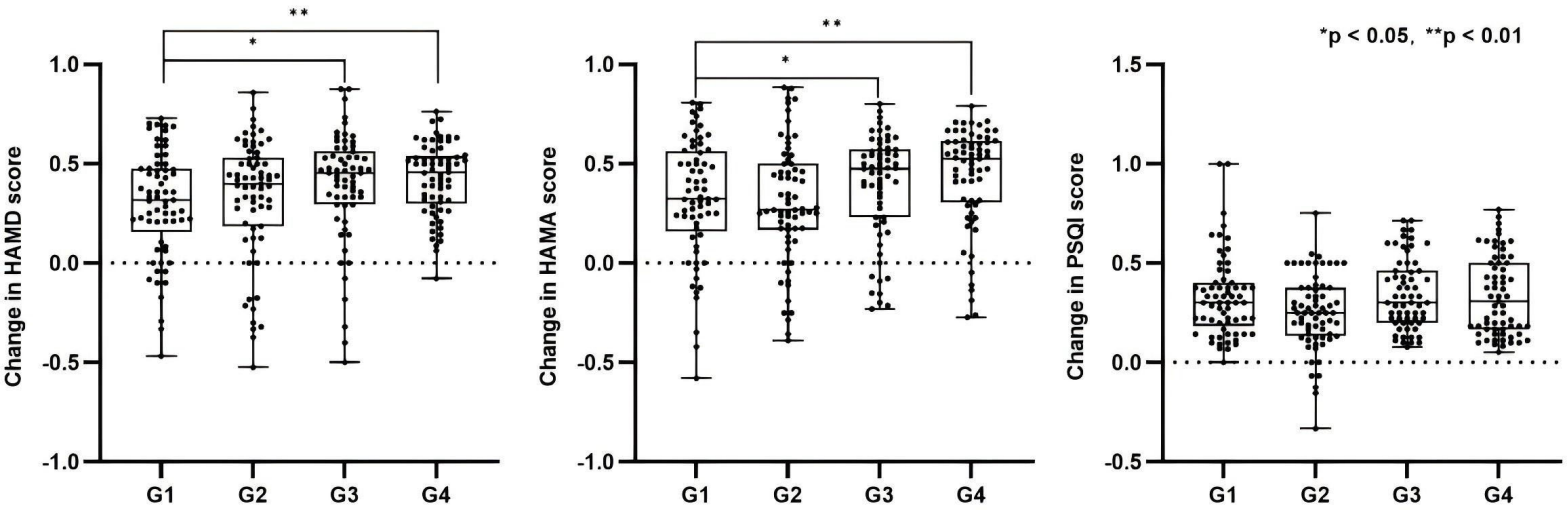
Reduction rates of HAMD, HAMA, and PSQI scores across groups and between-group comparisons. G1: medication only; G2: medication + tDCS; G3: medication + rTMS; G4: medication + tDCS + rTMS. *p < 0.05, **p < 0.01.

After 4 weeks of treatment, all four groups showed significant reductions in HAMD scores from baseline. The reduction rates in HAMD scores were as follows G1: 29.7%, G2: 32.8%, G3: 39.1%, and G4: 42.4% (**Table 2**). Mann-Whitney U tests revealed significant differences between G3 and G1 (*p* = 0.022), and between G4 and G1 (*p* = 0.006), indicating that combined rTMS or combined tDCS + rTMS treatment is more effective than medication alone in improving depressive symptoms. No significant differences were observed between the other groups (*p* > 0.05) (**Figure 2**).

**Table 2:** Reduction rates of HAMD, HAMA, and PSQI scores across groups.

Group	HAMD reduction rate (%)	HAMA reduction rate (%)	PSQI reduction rate (%)
G1	29.7 ± 27.1	32.1 ± 30.5	33.0 ± 20.6
G2	32.8 ± 30.6	29.9 ± 30.1	25.2 ± 19.0
G3	39.1 ± 27.8*	38.4 ± 26.7*	34.6 ± 17.8
G4	42.4 ± 18.1**	43.6 ± 25.7**	33.6 ± 19.9

G1: medication only; G2: medication + tDCS; G3: medication + rTMS; G4: medication + tDCS + rTMS. Data are presented as “mean ± standard deviation”; *p < 0.05, **p < 0.01, compared with G1, based on the Mann–Whitney U test.

### 3.3 Secondary endpoint

For anxiety symptoms, the reduction rates in HAMA scores were as follows: G1 32.1% ± 30.5%, G2 29.9% ± 30.1%, G3 38.4% ± 26.7%, and G4 43.6% ± 25.7% (**Table 2**). Mann–Whitney U tests revealed significant differences between G3 and G1 (*p* = 0.022), and between G4 and G1 (*p* = 0.006), indicating that combined brain stimulation interventions are more effective in alleviating anxiety (**Figure 2**).

Regarding sleep quality, the reduction rates in PSQI scores were as follows: G1 33.0% ± 20.6%, G2 25.2% ± 19.0%, G3 34.6% ± 17.8%, and G4 33.6% ± 19.9% (**Table 2**). Kruskal–Wallis test results showed no significant differences between the groups (*p* = 0.084) (**Figure 2**).

### 3.4 Analysis of factors influencing efficacy

The results of the regression analysis (**Table 3**) revealed that both baseline HAMD scores (OR = 1.081, 95% CI: 1.061–1.102, *p* < 0.001) and disease duration (OR = 0.960, 95% CI: 0.940–0.980, *p* < 0.001) were significantly associated with treatment efficacy. Specifically, a higher baseline HAMD scores were associated with a greater likelihood of achieving “effective improvement”, while shorter disease duration was associated with a higher probability of treatment efficacy. Other variables, including gender, age, BMI, and treatment group, did not reach statistical significance after adjusting for covariates (*p* > 0.05).

**Table 3:** Logistic regression analysis of factors influencing efficacy (HAMD reduction rate ≥25%).

Factor	OR	95% CI	*p*
Treatment Group (G2 vs G1)	1.19	0.48–2.95	0.71
Treatment Group (G3 vs G1)	1.81	0.78–4.18	0.18
Treatment Group (G4 vs G1)	2.46	0.94–6.45	0.07
Gender (Female vs Male)	0.853	0.658–1.106	0.23
Age (years)	1.026	0.973–1.081	0.34
Disease Duration (months)	0.96	0.94–0.98	<0.001
BMI	1.024	0.998–1.051	0.08
Baseline HAMD	1.081	1.061–1.102	<0.001

OR, Odds Ratio; CI, Confidence Interval.

### 3.5 Adverse events and safety

No serious adverse events were reported during the 4-week treatment period. Overall, patients in all groups demonstrated good tolerance, with adverse events being mild and mostly transient. Reported adverse events included dizziness (n = 6, 2.3%), scalp numbness (n = 7, 2.7%), nausea (n = 4, 1.5%), tremors (n = 5, 1.9%), dry mouth (n = 7, 2.7%), sweating (n = 3, 1.2%), drowsiness (n = 4, 1.5%), and one case of seizure (0.4%, in the G4 group). No patients experienced manic episodes or discontinued treatment due to adverse events (**Table 4**).

**Table 4:** Adverse events among patients receiving treatment.

Adverse event	G1 (n = 65)	G2 (n = 67)	G3 (n = 63)	G4 (n = 65)
Dizziness	1 (1.5%)	1 (1.5%)	2 (3.2%)	2 (3.1%)
Scalp tingling	0 (0.0%)	2 (3.0%)	2 (3.2%)	3 (4.6%)
Nausea	2 (3.1%)	1 (1.5%)	1 (1.6%)	0 (0.0%)
Tremor	1 (1.5%)	1 (1.5%)	2 (3.2%)	2 (3.1%)
Dry mouth	2 (3.1%)	1 (1.5%)	1 (1.6%)	2 (3.1%)
Sweating	1 (1.5%)	0 (0.0%)	1 (1.6%)	1 (1.5%)
Somnolence	0 (0.0%)	1 (1.5%)	1 (1.6%)	2 (3.1%)
Seizure	0 (0.0%)	0 (0.0%)	0 (0.0%)	1 (1.5%)

G1: medication only; G2: medication + tDCS; G3: medication + rTMS; G4: medication + tDCS + rTMS. Data are presented as n (%).

## 4 Discussion

This study demonstrates that the combination of rTMS and tDCS significantly enhances treatment efficacy in adolescents with depression compared with pharmacotherapy alone, compared to medication alone. No serious adverse events were reported, and all treatment regimens were well tolerated. This study extends previous findings to adolescents, suggesting that the sequential application of both neuromodulation techniques may produce a synergistic effect.

### 4.1 Mechanism of synergistic effect

The complementary nature of rTMS and tDCS, regarding their targets and mechanisms of action, may play a key role in the enhanced efficacy observed with their combined use. rTMS modulates emotion-related neural networks by applying high-frequency stimulation to the left dorsolateral prefrontal cortex (DLPFC), while tDCS influences neuronal excitability by applying weak electrical currents to adjust the membrane potential of neurons. When tDCS is applied first, it may “pre-activate” the cortex, thereby enhancing the effects of rTMS (16–18). Additionally, both rTMS and tDCS can induce cortical plasticity through different mechanisms (e.g., long-term potentiation-like processes and regulation of synaptic plasticity), and their combined use facilitates more profound functional remodeling within depression-related neural circuits (19). Several clinical meta-analyses have shown that the response rate to rTMS is generally higher than that of tDCS (7, 20, 21). However, when used together, tDCS and rTMS exhibit synergistic potential (15, 22).

Notably, the combined treatment group (G4) produced the greatest overall improvement in depressive and anxiety symptoms and was significantly superior to medication alone (G1). In contrast, tDCS alone (G2) showed no significant difference compared to medication monotherapy. This pattern prompts consideration of the specific contribution of tDCS within the combined protocol. Although the direct comparison between G4 and the rTMS-only group (G3) did not reach statistical significance, an additional 3.3-percentage-point mean reduction in G4, combined with the neurophysiological complementarity of rTMS and tDCS (see Section 4.1), is consistent with the hypothesis that tDCS may potentiate rTMS through cortical pre-conditioning. Definitive confirmation of this synergy—and quantification of tDCS’s independent contribution—will require adequately powered head-to-head trials directly comparing rTMS monotherapy with the rTMS + tDCS combination while incorporating concurrent neurophysiological assessments (e.g., EEG or fNIRS).

### 4.2 Analysis of baseline factors

The logistic regression analysis results indicated that baseline depressive symptom severity significantly influences treatment response. Several studies have shown that the more severe the baseline depression symptoms, the better the response to antidepressant medications. Furukawa et al. conducted a meta-analysis of individual patient data, highlighting that baseline depression severity moderates the efficacy of newer-generation antidepressants (23). The more severe the depression, the more pronounced the therapeutic benefit of antidepressants. Watanabe et al. also found that baseline characteristics, including the number of depressive symptoms and disease duration, predicted treatment response and remission more effectively than models relying solely on overall depression severity (24). Additionally, this study found that disease duration was significantly associated with efficacy, with shorter disease durations linked to a higher probability of achieving effective improvement. This result aligns with Khalid et al.’s study on treatment-resistant depression, where they noted that shorter disease duration is typically associated with better treatment outcomes (25). Taken together, baseline depression severity and disease duration are critical predictors of antidepressant treatment effectiveness.

### 4.3 Improvement of comorbid symptoms and safety

This study found that rTMS, particularly when combined with tDCS, demonstrated a significant advantage in alleviating comorbid anxiety and sleep disorders. Specifically, the reduction in HAMA scores was significantly greater in the rTMS and combined groups compared to the medication-only group, indicating that combined brain stimulation can effectively alleviate both depressive and anxiety symptoms. This finding aligns with a previous study by Vergallito et al., which found that combining rTMS and tDCS significantly improves both anxiety and depressive symptoms (26). No serious adverse events occurred during the treatment period, and all observed adverse reactions were mild and reversible. No patients experienced manic episodes or discontinued treatment due to adverse events, suggesting that rTMS combined with tDCS is safe and well tolerated in adolescents.

### 4.4 Evidence for rTMS in adolescent first-episode depression

Recent studies specifically examining rTMS in adolescents with first-episode MDD provide critical context for our findings. A randomized, double-blind, sham-controlled trial by Gu et al. (27) demonstrated that low-frequency rTMS (LF-rTMS) combined with medication was safe and well-tolerated in FE-MDD adolescents, with no negative impact on neurocognitive performance. While active LF-rTMS showed higher response (70.0% vs. 60.0%) and remission rates (55.0% vs. 35.0%) compared to sham stimulation, these differences did not reach statistical significance – highlighting the complex interplay of placebo effects and neuromodulation efficacy in this population. Further supporting rTMS safety, a systematic review by Zheng et al. (28) of three RCTs concluded that LF-rTMS could benefit drug-naïve FE-MDD adolescents without major safety concerns, though limited sample sizes constrained definitive efficacy conclusions. Crucially, a 2023 meta-analysis by Sun et al. (29) of six RCTs (n=562) found adjunctive rTMS (both high- and low-frequency) significantly improved depressive symptoms (SMD = -1.50) and response/remission rates versus controls (RR = 1.35 for both), while enhancing neurocognitive function in 80% of included studies. These collective findings support the notion that combined neuromodulation (rTMS + tDCS) is a viable strategy for adolescent depression. The synergistic efficacy observed in our G4 group aligns with Sun et al.’s robust evidence for adjunctive rTMS benefits.

### 4.5 Limitations and future research directions

This study introduces a new treatment strategy for adolescent depression, particularly for patients with poor responses to medication. rTMS can serve as an effective adjunctive treatment, and combining it with tDCS may further enhance therapeutic efficacy. However, this study has several limitations. First, although this was a randomized controlled trial, no *a priori* sample size calculation was performed. The sample size was determined based on all eligible and completed cases during the study period, consistent with a real-world pragmatic design. While the overall sample size is adequate, some subgroups within the treatment groups are relatively small, which limits the statistical power of subgroup analyses. Second, the follow-up duration was limited to 4 weeks, and long-term effects and relapse risk were not assessed. Future studies should extend the follow-up period to further assess the sustainability of treatment effects. Third, using reduction rates as the primary outcome may favor patients with higher baseline scores, potentially underestimating the improvements in those with milder depression. Future research should incorporate additional assessment measures, such as absolute score changes, to more comprehensively reflect treatment effects. Fourth, the study lacked a pure brain stimulation group, which prevented the independent effects of medication and brain stimulation from being quantified. Future studies should include a group receiving only brain stimulation to evaluate its effects separately. Additionally, the absence of a placebo group and the non-blinded interventions may have introduced expectancy effects. Future research should employ a double-blind, placebo-controlled design. In conclusion, future research should focus on increasing sample sizes, extending follow-up durations, and exploring the long-term effects and mechanisms of combined medication and brain stimulation treatments to further validate the stability and reliability of the findings.

## Data Availability

The original contributions presented in the study are included in the article/supplementary material. Further inquiries can be directed to the corresponding authors.
